# Vaccine-specific T-cell proliferation in response to a dual peptide cancer vaccine in breast and ovarian cancer patients

**DOI:** 10.1186/2051-1426-1-S1-P236

**Published:** 2013-11-07

**Authors:** Erika J Schneble, John S Berry, Alfred F Trappey, Timothy J Vreeland, Diane F Hale, Alan K Sears, Guy T Clifton, Sathibalan Ponniah, Nathan M Shumway, Elizabeth A Mittendorf, George E Peoples

**Affiliations:** 1 San Antonio Military Medical Center, San Antonio, TX, USA; 2 MD Anderson Cancer Center, Houston, TX, USA; 3 Uniformed Services University of Health Sciences, Bethesda, MD, USA; 4 Blanchfield Army Community Hospital, Fort Campbell, KY, USA

## Background

HER2 is a commonly expressed tumor-associated antigen in breast (BrCa) and, therefore, an attractive target for immunotherapy. We have investigated HER2-derived peptides as vaccines mixed with GM-CSF to include GP2 (a HLA-A2 and HLA-A3 restricted, CD8+ eliciting epitope) and AE37 (a HLA unrestricted, MHC class II, CD4+ eliciting epitope). Both peptide vaccines (PV) have shown clinical promise individually and there is clear rationale for combining GP2 and AE37 to elicit a more robust immune response (IR) of both CD4+ and CD8+ T cells. Here, we report the results of in vitro, CD8+, T-cell proliferation in response to the AE37 epitope.

## Methods

After completion of standard therapy, disease-free, node positive or high-risk, node negative BrCa or ovarian cancer patients received AE37/GP2+GM-CSF in six monthly intradermal inoculations (R1-R6). To determine HER2-induced proliferation, a 3H-thymidine incorporation assay was used. Specifically, peripheral mononuclear blood cells (PMBC) were isolated from blood samples taken at baseline, R3, R6, six months after the primary vaccine series (RC6), and 12 months post-PVS (RC12). PMBC were then exposed to the AE37 peptide or culture media (CM) as a control. After incubation with radioactive-thymidine and cell lysis, a scintillation counter was used to measure the amount of thymidine incorporation into DNA in counts per minute (cpm). Data are expressed as means +/-SEM and compared using student’s t-test.

## Results

Of the 28 vaccinated patients, PMBC from 22 patients were available. Data units are measured in cpm. Results are presented in the table below (Table 1).

**Table 1:** 

	R0	R3	R6	RC6	RC12
AE37	1023.9+216.7	2457.4+685.4	2170.3+461.6	3494.1+1442.5	2891.8+1542.8
CM	1405.9+260.6	1119.7+307.9	2027.2+682.8	1612.9+331.4	1370.1+367.8
p-value	0.57	0.02	0.06	0.11	0.35

## Conclusions

During the primary vaccination series, an AE37/GP2+GM-CSF dual peptide vaccine results in robust T-cell proliferation. However, significant immune responses become more variable at six and twelve months post vaccination suggesting the need for boosters in some individuals.

